# Synaptic Connections between Endomorphin 2-Immunoreactive Terminals and μ-Opioid Receptor-Expressing Neurons in the Sacral Parasympathetic Nucleus of the Rat

**DOI:** 10.1371/journal.pone.0062028

**Published:** 2013-05-03

**Authors:** Xiao Liang Dou, Rong Liang Qin, Juan Qu, Yong Hui Liao, Ya cheng Lu, Ting Zhang, Chen Shao, Yun Qing Li

**Affiliations:** 1 Department of Urology, Xijing Hospital, The Fourth Military Medical University, Xi'an, China; 2 Department of Anatomy, Histology and Embryology and K. K. Leung Brain Research Centre, The Fourth Military Medical University, Xi'an, China; The Research Center of Neurobiology-Neurophysiology of Marseille, France

## Abstract

The urinary bladder is innervated by parasympathetic preganglionic neurons (PPNs) that express μ-opioid receptors (MOR) in the sacral parasympathetic nucleus (SPN) at lumbosacral segments L6-S1. The SPN also contains endomorphin 2 (EM2)-immunoreactive (IR) fibers and terminals. EM2 is the endogenous ligand of MOR. In the present study, retrograde tract-tracing with cholera toxin subunit b (CTb) or wheat germ agglutinin-conjugated horseradish peroxidase (WGA-HRP) via the pelvic nerve combined with immunohistochemical staining for EM2 and MOR to identify PPNs within the SPN as well as synaptic connections between the EM2-IR terminals and MOR-expressing PPNs in the SPN of the rat. After CTb was injected into the pelvic nerve, CTb retrogradely labeled neurons were almost exclusively located in the lateral part of the intermediolateral gray matter at L6-S1 of the lumbosacral spinal cord. All of the them also expressed MOR. EM2-IR terminals formed symmetric synapses with MOR-IR, WGA-HRP-labeled and WGA-HRP/MOR double-labeled neuronal cell bodies and dendrites within the SPN. These results provided morphological evidence that EM2-containing axon terminals formed symmetric synapses with MOR-expressing PPNs in the SPN. The present results also show that EM2 and MOR might be involved in both the homeostatic control and information transmission of micturition.

## Introduction

The micturition reflex is an autonomic reflex that is mediated by a simple spino-bulbo-spinal pathway that passes through the pontine micturition center [1], [2], [3]. In rats, neurons in the sacral parasympathetic nucleus (SPN) at L6-S1 are involved in this reflex. Moreover, most of these neurons are located at the corresponding segments in the lateral part of the sacral intermediolateral gray matter [4], [5] and are known as parasympathetic preganglionic neurons (PPNs). PPNs send their axons to the pelvic organs *via* the pelvic nerve and are essential for the autonomic functions of the pelvic organs, such as the micturition reflex, defecation and sexual behavior [6], [7], [8]. It has been shown that electrical microstimulation of the SPN regions can induce bladder contraction [9], [10], [11], [12], [13].

Immunohistochemical studies have indicated that μ-opioid receptors (MOR)-immunoreactive (-IR) neurons are widely distributed in the spinal gray matter, particularly in the SPN [14], [15], [16]. These results are further supported by autoradiographic [17], [18] and in situ hybridization histochemical studies [19] in the spinal cord. Moreover, functional analyses have demonstrated that in the rat spinal cord, MOR agonists, such as morphine, the exogenous ligand of MOR, are involved in the inhibition of bladder control [20], [21], [22]. However, the mechanism underlying morphine inhibition on bladder contraction is still unclear. Thus, this inhibitory function is one of most crucial side-effects of morphine when it is used as an analgesic and limits its clinical usage. Because morphine is a common analgesic, it is important to overcome this disadvantage so that it can be used effectively in a clinical setting. Thus, it is critical to identify the mechanism underlying morphine inhibition of bladder control. Of all the known opioid substances, endomorphin 2 (EM2), an endogenous peptide ligand of MOR, exhibits the highest affinity for MOR [23], [24]. Previous studies have been conducted on the origins of EM2-IR fibers and terminals in the spinal cord. Using capsaicin-treatment or rhizotomy to disrupt the normal transportation and function of EM2 in primary afferents, these studies suggest that primary afferents are the major source of this opioid peptide [25], [26], [27], [28], [29], [30]. In addition, previous functional studies have also shown that opioid peptides might inhibit the micturition reflex at the spinal level [31].

On the basis of these results, we propose the hypothesis that within the SPN, EM2 released from primary afferents inhibit the activities of MOR-expressing PPNs via its binding to MOR in PPNs. Consequently, this reduces the activities of the PPNs, and results in inhibitory effects on the micturition reflex. To confirm this hypothesis and to reveal the mechanism underlying morphine inhibition of bladder control, the present study was designed to examine the direct connections between EM2-IR primary afferent terminals and MOR-IR PPNs in the SPN using cholera toxin subunit b (CTb) retrograde tracing combined with immunohistochemical staining of EM2 and MOR to identify PPNs and to visualize the synapses between EM2-IR terminals and MOR-IR PPNs in the SPN.

## Materials and Methods

A total of 40 adult male *Sprague-Dawley* rats (180–250 g) were used for the present experiments. The animals were provided by the Experimental Animal Center of the Fourth Military Medical University (Xi'an, China). All of the protocols were approved by the Animal Care and Use Committee at the Fourth Military Medical University and were performed in accordance with the animal care rules set forth by the university (Permit number: 10001). All efforts were made to reduce the number of animals used and to minimize their suffering.

### Retrograde tract-tracing using cholera toxin subunit b (CTb)

Ten rats were anesthetized with a 2% sodium pentobarbital solution (40 mg/kg, *i.p.*). The surgical procedure was performed under an operating microscope. The left pelvic nerve of each animal was exposed via a posterior approach through the sacrococcygeal region. The nerve was then completely transected at a distance of 1 mm proximal to the major pelvic ganglion. An 8-mm-long polyvinylchlorid (PVC) pipe with an inner diameter of 1 mm was prepared, and one end was closed by heating. Next, a microsyringe was used to infuse 2–3 µl of 10% Alexa 594-conjugated CTb (C34777; Invitrogen, Carlsbad, CA, USA) that had been dissolved in 0.1 M PB (pH 7.4) into the pipe. After the central end of the pelvic nerve was placed into the pipe, the opening of the pipe was plugged using super glue and any excess CTb from the outside of the pipe was removed. The pipe was then fixed to the nerve stump *in situ*, and the wound was sutured. The rats were allowed to survive for 5 days following pelvic nerve retrograde tracing. After 5 days, the rats were anesthetized with an overdose of 2% (w/v) pentobarbital sodium (100 mg/kg), which was administered *via* intraperitoneal injection (*i.p.*), and all of the rats were transcardially perfused with 100 ml of 0.01 M phosphate-buffered saline (PBS, pH 7.4) followed by approximately 500 ml of a 0.1 M phosphate buffer (PB, pH 7.4) that contained 4% (w/v) paraformaldehyde and 75% (v/v) saturated picric acid. The lumbosacral cords were then rapidly dissected, removed, and divided into L6-S1 segments. The segments were postfixed for 6–8 h at 4°C and subsequently placed in a 0.1 M PB solution containing 30% (w/v) sucrose for 24 h at 4°C prior to being embedded in an inert mounting medium (OCT; Tissue-Tek; Sakura; Torrance, CA, USA). Thirty-µm thick transverse sections of the L6-S1 segments were obtained from 5 of the 10 rats and the distribution of the CTb retrogradely labeled neurons in the SPN were examined. In addition, longitudinal sections with the same thickness and of similar segments were obtained from the remaining 5 rats and the spatial structure of the SPN was analyzed. During the sectioning process, 5 groups of transverse sections from each rat were collected into 5 dishes and 5 groups of longitudinal sections from another rat were collected into a different set of 5 dishes. All of the dishes contained 0.01 M PBS (pH 7.4), and each dish contained every fifth serial transverse or longitudinal section. Next, the sections in the first dish were mounted onto gelatin-coated glass slides, air dried and coverslipped with a mixture of 50% (v/v) glycerin and 2.5% (w/v) triethylene diamine (anti-fading agent) in 0.01 M PBS. Finally, the sections were observed using a fluorescence microscope (Olympus BX-60; Tokyo, Japan).

### Immunofluorescence histochemical staining of MOR and EM2

The rats (*n* = 10) were anesthetized and perfused as previously described. Next, the L6-S1 spinal segments were removed, postfixed and cut into 30-µm thick transverse sections and divided into 6 groups. Each group contained every sixth serial section. The sections were then blocked for 30 min with 10% fetal calf serum (FCS) in PBS (0.01 M, pH 7.4). Next, the sections in the first and second groups were processed for EM2 or MOR immunofluorescence histochemical staining, respectively. The sections were subjected to the following series of incubations: (1) incubation with rabbit antiserum against EM2 (1∶200; AB10289; Abcam, Cambridge, MA, USA) or guinea pig antiserum against MOR (1∶1000; AB1774; Millepore, Billerica, MA, USA) in the antibody dilution medium for 72 h at 4°C. The medium consisted of 0.01 M PBS (pH 7.4) containing 5% (v/v) normal donkey serum (PBS-NDS), 0.3% (v/v) Triton X-100, 0.05% (w/v) NaN_3_ and 0.25% (w/v) λ-carrageenan; (2) incubation with biotinylated donkey anti-rabbit IgG (1∶500; AP182F; Millipore) or biotinylated goat anti-guinea pig IgG (1∶500; BA-7000; Vector, Burlingame, CA, USA) in PBS-NDS for 12 h at 4°C; and (3) incubation with fluorescein isothiocyanate (FITC)-labeled avidin D (1∶1000; A-2001; Vector) in PBS containing 0.3% Triton X-100 (PBS-X, pH 7.4) for 2 h at room temperature (RT; 24±3°C). The sections were washed with PBS containing 0.3% Triton X-100 (PBS-X, pH 7.4), and sections that had been processed for immunofluorescent staining for MOR and EM2 were mounted, cover-slipped and examined using a confocal laser scanning microscope (Olympus FV1000; Tokyo, Japan).

The sections in the third group were processed for immunofluorescence histochemical double labeling for EM2 and MOR. Briefly, the sections were sequentially incubated with: (1) a mixture of rabbit antiserum against EM2 (1∶200; AB10289; Abcam) and guinea pig antiserum against MOR (1∶1000; AB1774; Millipore) in PBS-NDS for 72 h at 4°C; (2) biotinylated donkey anti-rabbit IgG (1∶500; AP182F; Millipore) for 12 h at 4°C; and (3) FITC-labeled avidin D (1∶1000; A-2001; Vector) and Alexa 594-labeled goat anti-guinea pig IgG (1∶500; A-11076; Invitrogen) for 2 h at RT. The sections were then mounted, cover-slipped and examined using the confocal laser scanning microscope (Olympus FV1000).

The sections of the remaining 3 groups were used as controls. In the control studies, the sections in the fourth, fifth and sixth groups were incubated with normal rabbit serum, normal guinea pig serum and a mixture of normal rabbit and guinea pig sera diluted with PBS-NDS, respectively. The procedures and reagents used in the control tests were similar to those previously described. There were no immunopositive staining results observed on the control sections.

### CTb retrograde tracing combined with immunofluorescence histochemical staining for EM2 and MOR

A total of 10 rats were used for the triple-labeling experiment. These rats were subjected to CTb retrograde tract-tracing, perfused and then sectioned using the same protocols as previously described. Thirty-micron-thick transverse sections of the L6-S1 spinal segments were collected into two dishes in cold PB (0.01 M, pH 7.4), where each dish contained a series of every second serial section. The sections in the first dish were then processed for immunofluorescence histochemical triple labeling for EM2, MOR and CTb. These sections were initially blocked with 10% FCS in PBS (0.01 M, pH 7.4) for 30 min, after which the sections were sequentially incubated in the following: (1) a mixture containing rabbit antiserum against EM2 (1∶200; AB10289; Abcam), guinea pig antiserum against MOR (1∶1000; AB1774; Millipore) and goat antiserum against CTb (1∶500; 7032A6; Biological, Massachusetts, MA, USA) diluted in PBS-NDS for 72 h at 4°C; (2) biotinylated donkey anti-rabbit IgG (1∶500; AP182F; Millipore) in PBS-NDS for 12 h at 4°C; and (3) a mixture containing FITC-labeled avidin D (1∶1000; A-2001; Vector), Alexa 647-labeled donkey anti-guinea pig IgG (1∶500; AP193SA6; Millipore) and Alexa 594-labeled donkey anti-goat IgG (1∶500; A-11058; Invitrogen) in PBS-X (pH 7.4) for 2 h at RT.

The sections in the second dish were used as controls. For the controls, the primary antibodies were replaced with a mixture of normal rabbit, guinea pig and goat sera. The incubation medium and reagents used for each group were prepared as previously described. Next, the sections were mounted, cover-slipped and examined using a confocal laser scanning microscope (Olympus FV1000).

### Triple-labeled electron microscopy revealed synaptic connections between EM2-immunopositive terminals and WGA-HRP retrogradely labeled neurons expressing MOR in the SPN

Ten male *Sprague-Dawley* rats were used in the following experiment. The central cut ends of the pelvic nerves of the rats were injected with 0.2–0.4 µl of 20% (w/v) wheat germ agglutinin-conjugated horseradish peroxidase (WGA-HRP; Toyobo, Tokyo, Japan), which is used as a retrograde tracer. The procedures are similar to CTb retrograde tract-tracing in the light microscopy study. The rats survived for 4 d following the WGA-HRP retrograde tracing and were then deeply anesthetized and transcardially perfused with 100 ml of 0.01 M PBS (pH 7.4) followed by 500 ml of 0.1 M PB (pH 7.4) containing 4% (w/v) paraformaldehyde, 0.1% (w/v) glutaraldehyde, and 15% (v/v) saturated picric acid. All of the solutions used for the tissue perfusion were maintained at 4°C. After the perfusions, the L6-S1 segments were immediately removed from each animal and cut into 50-µm transverse sections using an Oscillating Tissue Slicer (DTK-1000; Dosaka, Kyoto, Japan). The sections were subsequently divided into two groups, where each group contained a series of alternating serial sections. The sections were collected in 0.01 M PBS (pH 7.4) and treated with tetramethylbenzidine (TMB) for WGA-HRP histochemical staining. Sodium tungstate was used as a stabilizer, and the reaction products were intensified using a 3,3-diaminobenzidine tetrahydrochloride (DAB)/cobalt/H_2_O_2_ solution [32]. The sections were then mounted on to glass slides, and the distribution of WGA-HRP-labeled neurons in the SPN was examined using a light microscope. Sections containing WGA-HRP-labeled neurons were selected for further study. These sections were cryoprotected in 10%, 20% and 30% sucrose in 0.05 M PB that contained 10% (v/v) glycerol for 30 min, and then freeze-thawed with liquid nitrogen to enhance the degree of antibody penetration.

After 30 min of blocking with 10% FCS in PBS (0.01 M, pH 7.4), immunoperoxidase and immunogold-silver methods were used to label EM2 and MOR proteins. Briefly, the sections were incubated with 0.05 M Tris-HCl buffered saline (TBS, pH 7.4) containing 20% (v/v) normal fetal calf serum for 1 h at RT to block nonspecific immunoreactivity. The sections were then incubated with a mixture of rabbit antiserum against EM2 (1∶100; AB10289; Abcam) and guinea pig antiserum against MOR (1∶1000; AB1774; Millipore) diluted in TBS containing 2% (v/v) normal donkey serum (TBS-D) for 72 h, washed in 0.05 M TBS and subjected to an overnight incubation with a mixture of 1∶500-diluted biotinylated anti-rabbit IgG (AP182F; Millipore) and 1∶100-diluted 1.4-nm gold particle-conjugated anti-guinea pig IgG (2055; Nanoprobes) in TBS-D. After postfixing the sections with glutaraldehyde and several washes in distilled water, the sections were subjected to silver enhancement using an HQ Silver Kit (2012; Nanoprobes) in the dark, followed by a subsequent 2 h incubation with an ABC Kit (Vector). Finally, the sections were placed in a 0.05 M Tris-HCl (pH 7.5) solution containing 0.02% DAB (Dojin, Kumamoto, Japan) and 0.003% H_2_O_2_ for 25–30 min. The immunolabeled sections were then postfixed in 1% OsO4, counterstained with 1% uranyl acetate in 70% ethanol, dehydrated, flat-embedded in Durcupan (Fluka, Buchs, Switzerland) and polymerized. Small pieces of the SPN regions that contained a large number of WGA-HRP retrogradely labeled neuronal cell bodies and axon terminals were selected and removed from the flat-embedded sections under a dissection microscope [33]. The selected tissue pieces were then cut into serial, ultrathin sections with an ultratome (Reichert-Nissei Ultracut S; Leica, Wien, Austria) mounted on to single-slot grids that had been coated with pioloform membrane, stained with 1% lead citrate, and finally examined with an electron microscope (CM100; Philips, Eindhoven, Netherlands).

### Microscopic observations

The sections for immuonfluorescence histochemical staining were observed under a confocal laser scanning microscope with the appropriate filters for FITC (excitation 492 nm; emission 520 nm), Alexa 647 (excitation 647 nm; emission 666 nm) and Alexa 594 (excitation 590 nm; emission 618 nm), respectively. All of the Alexa 594 conjugated CTb retrogradely traced sections were observed under a fluorescence microscope with the appropriate filters for CTB-labeled neurons (excitation 590 nm; emission 618 nm). The synaptic connections between the EM2-immunopositive terminals and WGA-HRP retrogradely labeled neurons expressing MOR in the SPN were examined under an electron microscope.

## Results

### Distribution and morphological features of CTb-labeled neurons in the SPN

Previous studies have demonstrated that sacral parasympathetic preganglionic neurons (PPNs) in the sacral parasympathetic nucleus (SPN) can be assigned to 3 groups on the basis of their morphology and location [7], [34]. These groups are denoted as the dorsal band (DB), lateral band (LB) and internal band (IB). The LB is located just above the DB, and these two groups of neurons are separated by the IB, which contains several isolated neurons [7], [34].

**Figure 1:**
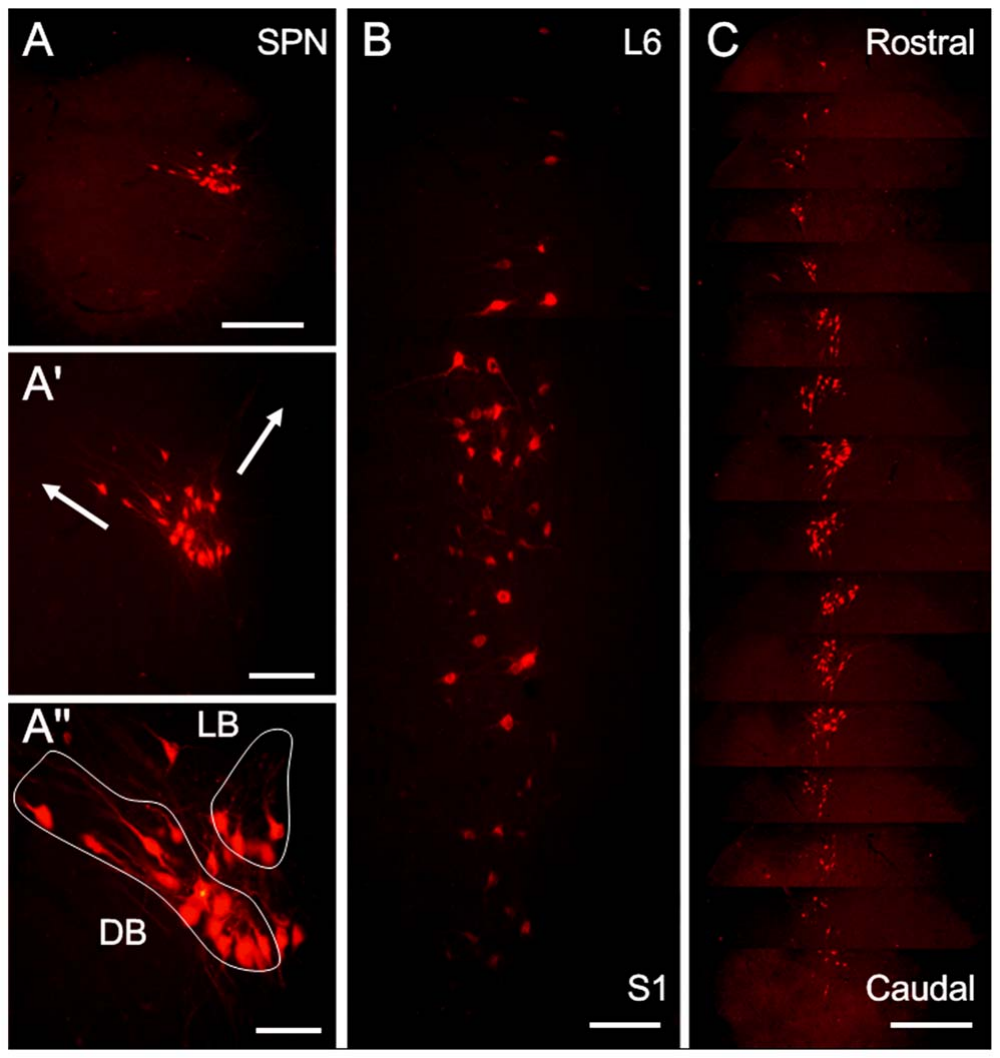
The distribution patterns of CTb labeled PPNs in the SPN at L6-S1 after CTb was injected into the left pelvic nerve. (A) The location of the CTb-labeled neurons in the SPN at S1. (A′) An enlarged image of (A), showing the extending directions of the dendrites of the CTb-labeled neurons in the lateral band (LB) or dorsal band (DB) (arrows); A″ is also an enlarged image of A, in which CTb-labeled neurons in the SPN belong to 3 groups: DB, LB and internal band (IB) (outside of the two circles). In the longitudinal sections (B), CTb-labeled neuronal clusters in the SPN resemble a “string of beads.” C: CTb-labeled neurons in the SPN in one rat are arranged in a sequence from the rostral to caudal levels of the nucleus. Scale bars indicate 200 µm in A and C, 100 µm in A′ and B and 50 µm in A″.

In the present study, after CTb injection into the left pelvic nerve, CTb retrogradely labeled PPNs were almost exclusively found in the lateral edge of the intermediolateral column in the left gray matter at L6-S1 (Figure 1A). These CTb-labeled neurons were generally located in or near the intermediolateral nucleus. In transverse sections, CTb-labeled neurons could be clearly divided into three groups. The LB group was located in the dorsolateral portion of the SPN and contained neurons of various shapes (Figure 1A″), including spindle-shaped neurons, rotund neurons and triangular neurons. Dendrites of the neurons in this band extended along the lateral marginal zone of the dorsal horn and into the dorsolateral funiculus (Figure 1A′). In contrast, the DB group was located deeper within the spinal cord and comprised of radially oriented neurons with dendrites that extended medially into the dorsal gray commissure (Figure 1A′). The distributions of the CTb-labeled neurons in the longitudinal (Figure 1B) and transverse (Figure 1C) sections further elucidated the columnar structure of the CTb-labeled PPNs in the SPN, which consisted of neuronal cell clusters that resembled “strings of beads.” The clusters had an average length of 2850±120 µm. In the ipsilateral SPN at L6-S1, approximately 630±21 medium-sized CTb retrograde-labeled neurons, of which the sizes ranged from 8–13 µm (shorter axis) to 10–22 µm (longer axis), were observed in each rat. The number of CTb retrograde-labeled neurons dramatically increased between the middle section of L6 and the middle section of S1, reaching its peak in the upper and middle parts of S1, and then gradually decreased until it disappeared completely in the lower part of S1.

### Immunofluorescence histochemical localization of MOR immunoreactivity in the SPN

**Figure 2:**
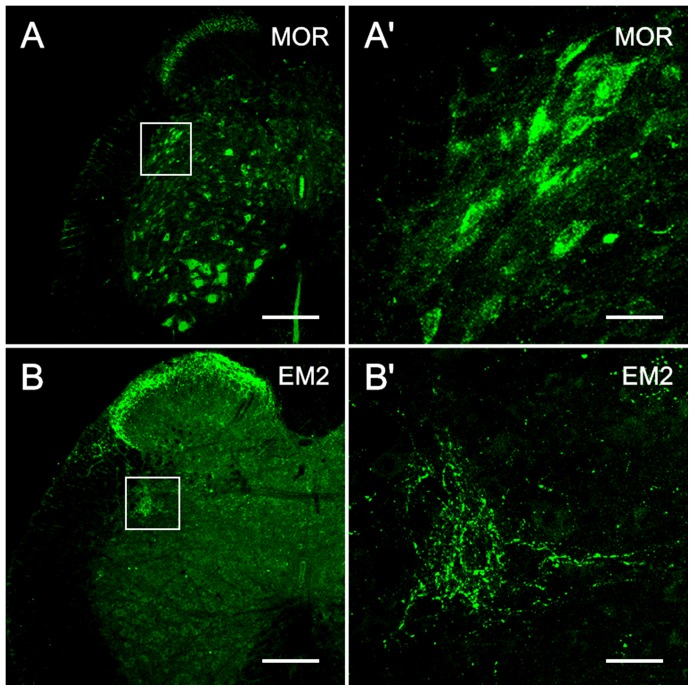
MOR and EM2 immunoreactivity in the SPN. The localization of MOR-immunoreactivity at S1 (A, A′) and EM2-immunoreactivity at L6 (B, B′) in the SPN on transverse sections. A, B: low magnification image. A′, B′: magnified image of the SPN as demarcated with a rectangle in A and B. Many neuronal cell bodies and their dendritic processes in the SPN show MOR-immunopositive staining (A′) and many EM2-IR fibers and terminals are present in the SPN (B′). The scale bars indicate 200 µm in A and B, 30 µm in A′ and B′.

**Figure 3:**
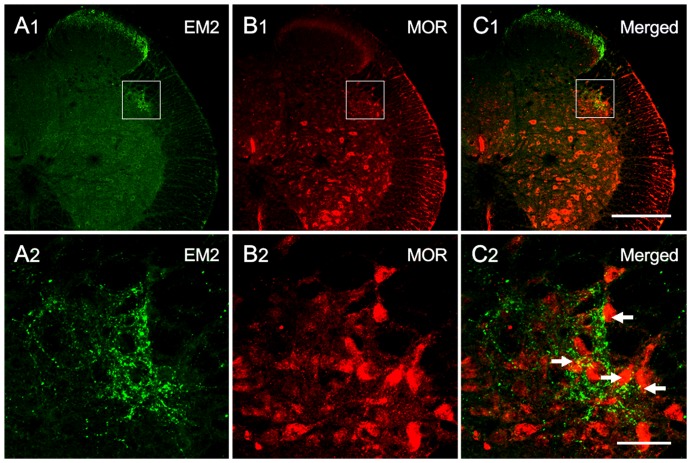
Immunofluorescence double labeling of EM2 and MOR immunoreactivity in the SPN on transverse sections at S1. Low-magnification images of the immunofluorescence histochemical staining for EM2 (A1) and MOR (B1) in the SPN (indicated with squares). Scattered EM2-IR fibers and terminals (A2) and many MOR-IR neurons (B2) are observed in the SPN. EM2-IR axon terminals also appear to be closely apposed with the MOR-IR neurons (indicated with arrows) in the SPN (as indicated with a square in C1) (C2). The scale bars indicate 200 µm in A1, B1 and C1 and 40 µm in A2, B2 and C2.

Many MOR-immuonreactive (-IR) neurons and their processes were observed in the intermediolateral cell column, *i.e.*, SPN (Figures 2A, 3B1). This expression pattern was similar to that described in a previous report [14]. In the SPN, MOR-IR products were widely distributed throughout all of the PPNs. These MOR-immunopositive neuronal cell bodies exhibited similar morphological features and their range in diameter was similar to that of CTb-labeled PPNs in the SPN at L6-S1. Most of these neurons were fusiform, triangular, or multipolar in shape; and were small to medium in size (ranging from 8–13 µm along the shorter axis and 10–22 µm along the longer axis) (Figures 2A′, 3B2). Intense MOR immunoreactivity was primarily observed in the neuronal cell bodies and their processes (Figures 2A′, 3B2).

### Distribution of EM2 immunoreactivity in the SPN

The EM2-IR structures in the SPN were fibers and terminals (Figures 2B′, 3A2). Most of the EM2-IR products in the SPN were diffusely fine, punctuate and granular in appearance (Figure 2B′). A higher density of EM2-IR fibers and terminals were observed in close proximity to the neuronal cell bodies located in the lateral band (LB) compared to the dorsal band (DB) (Figures 2B′, 3A2). No EM2-IR neuronal cell bodies were observed in the SPN as assessed using EM2 immunofluorescence histochemical staining.

### Synaptic connections between EM2-IR fibers and MOR-IR neurons in the SPN

**Figure 4:**
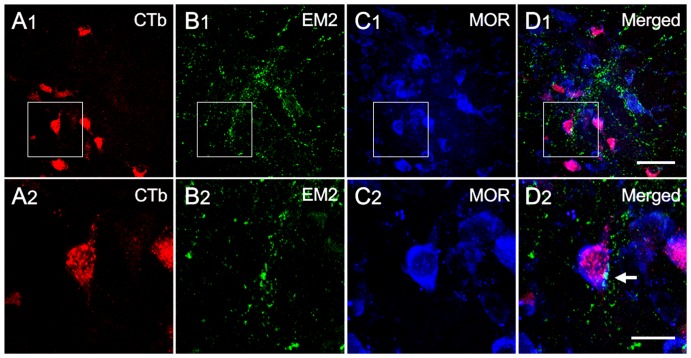
The connections between EM2-IR axon terminals and CTb/MOR double-labeled neurons in the SPN on a transverse section at S1. A2, B2, C2 and D2 are enlargements of the rectangles delineated on A1, B1, C1, D1, respectively. After injecting CTb into the pelvic nerve, CTb-labeled neurons were found in the SPN (A1, A2). Several CTb-labeled neurons contacted EM2-IR fibers and terminals (B1, B2; D1, D2) and also exhibited MOR-immunoreactivity (C1, C2; D1, D2). The white arrow points to suggestive close appositions between EM2-IR axon terminals and a CTb/MOR double-labeled neuron. The scale bars indicate 50 µm in A1, B1, C1 and D1 and 20 µm in A2, B2, C2 and D2.

On preparations exhibiting double immunofluorescence labeling, EM2-IR axon terminals appeared to be closely apposed with MOR-IR neuronal cell bodies and their dendritic processes in the SPN (Figures 3C1, C2). To provide morphological confirmation that the MOR-IR neurons were PPNs, immunofluorescence histochemical triple-labeling was performed. Initially, neurons in the SPN were successfully identified using CTb retrograde transport tracing (Figures 4A1, A2). We observed that all of the CTb-labeled neurons demonstrated MOR-IR staining (Figures 4C1, C2), and the EM2-IR fibers and terminals were distributed throughout the SPN (Figures 4B1, B2). Moreover, we observed beaded-like EM2-IR fibers with varicosities (Figures 3A2, 4B2), and the EM2-IR terminals appeared to be closely apposed with the CTb-labeled neurons, which were MOR-immunopositive in the SPN (Figures 4D1, D2).

**Figure 5:**
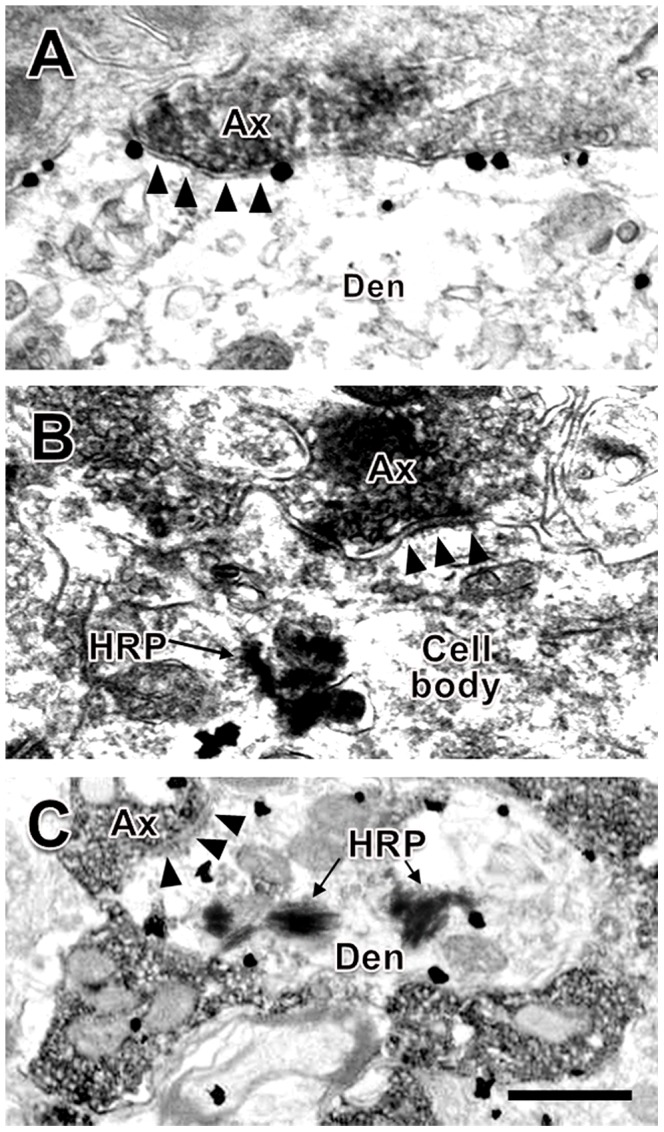
Synaptic connections between EM2-IR axon terminals (containing DAB reaction products) and HRP-labeled neuronal cell body (containing TMB reaction products), MOR-IR (containing gold-silver grains) or HRP/MOR double-labeled dendrites (containing both TMB reaction products and gold-particles) of the PPNs in the SPN. Three EM2-IR axons made symmetric synaptic connections with an MOR-IR dendrite (A), an HRP retrograde-labeled neuronal cell body (B) and an HRP/MOR double-labeled dendrite (C) of the PPNs in the SPN. The scale bar indicates 0.3 µm in A–C.

Electron microscopy was used to confirm the existence of synaptic connections between the EM2-IR axon terminals and MOR-IR neurons, WGA-HRP-labeled neurons or WGA-HRP/MOR double-labeled neurons in the SPN (Figure 5). Using electron microscopy, the presence of heavily stained and predominantly homogeneous black substances (DAB reaction products) were distributed in the axoplasm and around synaptic vesicles (Figures 5A, B, C). After enhancement with the HQ Silver Kit, immunogold-labeling of black oval or round particles with high electron densities were found underneath the plasma membrane of the neuronal cell bodies and large dendritic processes (Figures 5A, C). In the present study, DAB reaction products, immunogold particles and TMB reaction products were used to label the EM2-IR axon terminals (Figures 5A, B, C), MOR-IR neurons (Figures 5A, C) and WGA-HRP retrograde-labeled neuronal cell bodies and their processes (Figures 5B, C), respectively.

In the present study, EM2-IR axon terminals formed symmetric synaptic connections with MOR-IR dendritic processes (Figure 5A, C) and cell bodies, WGA-HRP-labeled neuronal cell bodies (Figure 5B) and WGA-HRP-labeled dendritic processes or WGA-HRP/MOR double-labeled neuronal cell bodies anddendritic processes (Figure 5C). Of all synapses made between EM2-IR axon terminals and WGA-HRP/MOR double-labeled neuronal cell bodies and their dendritic processes, 93% (28/30) were axo-dendritic synapses (Figures 5C) and only 7% (2/30) were axo-somatic synapses. No asymmetric synapses were observed between the EM2-IR axon terminals and MOR-IR neurons or WGA-HRP/MOR double-labeled neurons.

## Discussion

Micturition is a complex behavior involving cortical [35], subcortical [36], brainstem [2], [3], [37], spinal cord [38] and bladder mechanisms [39], [40]. In the spinal cord, neurons involved in the regulation of micturition are located in the superficial dorsal horn [41], dorsal gray commissure [1], Onuf's nucleus [42] and sacral parasympathetic nucleus (SPN) [7], [34]. In rats, parasympathetic preganglionic neurons (PPNs) in the SPN play an important role in micturition [6]. This study indicated that all of the PPNs in the rat SPN are MOR-immunopositive, which is consistent with the results of previous studies in the spinal cord using autoradiographic [17], [18], cystometrographic [43] and immunohistochemical [44] methods. In addition, we also demonstrated that EM2-containing fibers and terminals were both distributed within and adjacent to the SPN. Our previous results indicated that there were 3 potential origins for the EM2-containing fibers and terminals in the spinal cord: projection fibers from superior structures, such as the solitary tract nucleus and hypothalamus, dorsal horn neurons and primary afferent terminals. However, the major source of EM2-containing fibers and terminals in the spinal cord is the ipsilateral primary afferent fibers [25], [30]. Together with previous studies, which have demonstrated that numerous primary afferents from the bladder project to the sacral parasympathetic nucleus [7], [45], we conclude that the primary sensory afferents from the bladder are important contributors of EM2-containing terminations onto PPNs in the SPN region.

The present study shows that EM2 axon terminals form close connections with MOR-expressing PPNs. Within the SPN, PPNs may be divided into 3 groups, the LB, DB and IB, on the basis of different characteristics, including the location within the nucleus, the direction of dendritic conduction and morphological features [7]. It has also been demonstrated that the function of these neurons in each part of the SPN is different. Neurons in the LB in lamina VII provide inputs to the bladder detrusor; neurons in the BD in laminae V and VI provide inputs to the intestine; most of the neurons in the IB, which are located between the DB and LB, are interneurons and indirectly correlate with the activity of the intestine and urinary bladder [7], [34]. In the present experiments, we observed closer connections between the EM2-containing axon terminals and MOR-expressing neurons in the LB, which provide the major innervations to the bladder detrusor compared to the DB and IB. Electron microscopy studies have further shown that EM2-containing axon terminals form symmetric synapses with the cell bodies and dendritic processes of PPNs which exhibit both MOR-immunopositive staining and WGA-HRP retrograde labeling. These results suggest that EM2 might have more profound effects on LB neurons than neurons in the DB and IB. It is already well accepted that symmetric synapses are inhibitory synapses [46], so the function of the synaptic structures can be predicated by their morphological features. Taken together, our present data providemorphological evidence to support our hypothesis that EM2 released from primary afferent fibers inhibit the activity of MOR-expressing PPNs. In the rat, MOR are known to be involved in central opioid modulation of bladder motility at both the supraspinal and spinal sites [20], [46], [47], [48]. In the rat spinal cord, both pharmacological and behavioral studies have provided compelling evidence that inhibition of micturition is mediated by MOR [31], [49], [50]. Bladder contractions may be inhibited by the MOR exogenous agonist morphine in an isovolumetric rat model, and this effect is abolished by the intravenous administration of the MOR antagonist naloxone [21], further suggesting that MOR in the spinal cord is involved in the regulation of bladder function. The EM2 effects on micturition are similar to those of morphine [20], [51] and other opioid peptides [31], [52], [53], [54], resulting in the inhibition of the micturition reflex and subsequent urinary retention. Upon EM2 binding to MOR, the interactions between the ligand-receptor may occur via the release of the peptide and the subsequent activation of a postsynaptic MOR, resulting in inhibition of MOR-expressing PPNs in the SPN.

**Figure 6:**
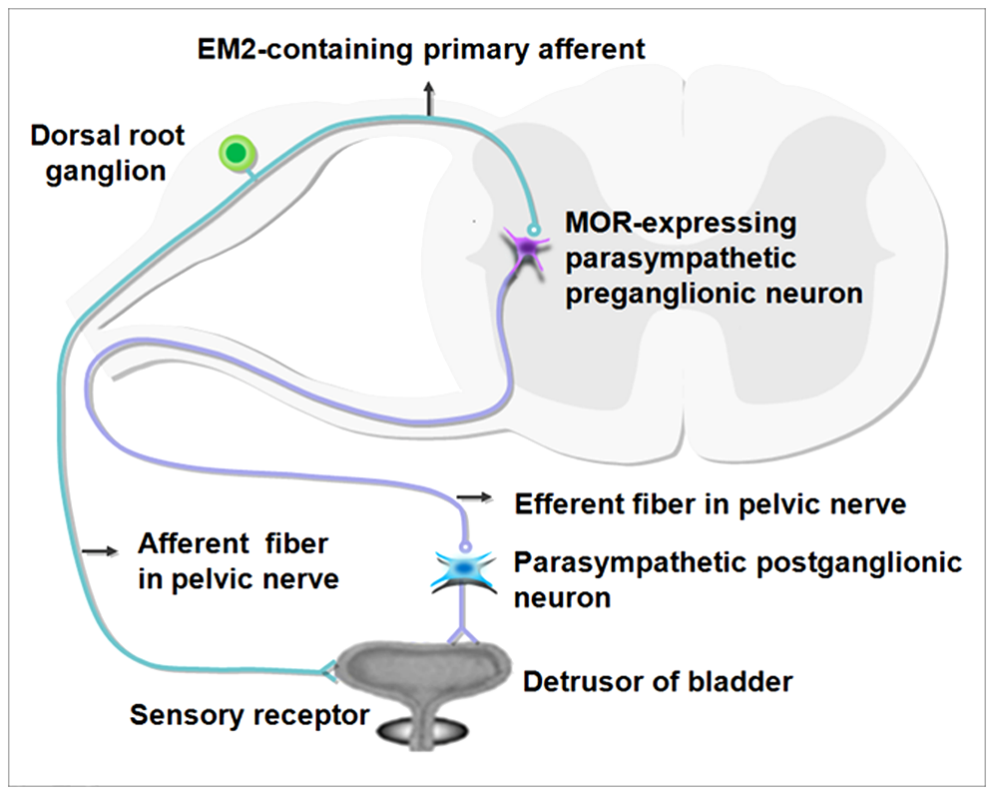
A schematic drawing showing the underlying mechanism of micturition inhibition by EM2-containing bladder afferent terminals onto the MOR-expressing PPNs in the SPN. Sensory information from the bladder receptor is directly transmitted to the MOR-expressing PPNs *via* EM2-containing primary afferent fibers. These EM2-containing fibers exert inhibitory effects onto the MOR-expressing PPNs in the SPN via synaptic connections, resulting in an attenuation of the excitatory effects of the efferent fibers originating from the PPNs onto the parasympathetic postganglionic neurons in the pelvic ganglia. This attenuation affects the micturition reflex and results in urinary retention.

Behavioral experiments and cystometry in the bladder have measured the effects of exogenous MOR agonists [55], [56], [57]. These studies have shown that in vitro activation of MOR reduces the contraction of the detrusor [58], [59]. However, the potential local and direct effects of morphine on bladder activity in the spinal cord have been less clear. In the present study, EM2, an endogenous agonist of MOR [60], was used as a substitute for the exogenous MOR agonist to explore the underlying mechanisms of urinary disorders caused by morphine. Our results showed that direct synaptic connections between EM2-containing terminals and MOR-expressing PPNs exist in the SPN. Information of the bladder travels via afferent fibers within the pelvic nerve into the lumbosacral spinal cord. In the SPN, these EM2-containing fibers form symmetric synaptic connections with MOR-expressing PPNs. EM2 is released from the presynaptic bouton and binds with MOR in the postsynaptic membrane, resulting in the inhibition of PPNs activity. Thus, the excitatory information transmitted via the parasympathetic preganglionic efferent fibers to the parasympathetic postganglionic neurons, which innervate the detrusor of bladder in the pelvic ganglion, will be reduced, resulting in a significant attenuation in the contractions in the rat bladder (Figure 6). Finally, the micturition reflex will be affected, which results in urinary retention.

## Conclusions

Taken together, the results of the present study show that there are direct symmetric synaptic connections between EM2-containing primary afferent terminals and MOR-expressing parasympathetic preganglionic neurons (PPNs) in the SPN. These synaptic connections further confirm our hypothesis that EM2 might act via MOR-expressing PPNs within the SPN and contributes to the inhibitory effects on bladder control. Thus, we infer that the inhibition of EM2-containing primary afferent terminals to MOR-expressing PPNs is partially derived from the synaptic mechanisms.

## References

[1] Blok BF, Holstege G (1998) The central nervous system control of micturition in cats and human. Behav Brain Res 92 (5) 119–125. 9638954 10.1016/s0166-4328(97)00184-8

[2] Ding YQ, Zheng HX, Gong LW, Lu Y, Qin BZ, et al (1997) Direct projections from the lumbosacral spinal cord to Barrington's nucleus in the rat: a special reference to micturition reflex. J Comp Neurol 389 (1) 149–160. 9390766 10.1002/(sici)1096-9861(19971208)389:1<149::aid-cne11>3.0.co;2-g

[3] Kruse MN, Mallory BS, Noto H, Roppolo JR, De Groat WC (1992) Modulation of the spinobulbospinal micturition reflex pathway in cats. Am J Physiol 262 (3) 478–484. 10.1152/ajpregu.1992.262.3.R478 1558219

[4] Hancock MB, Peveto CA (1979) Preganglionic neurons in the sacral spinal cord of the rat: an HRP study. Neurosci Lett 11 (1) 1–5. 86176 10.1016/0304-3940(79)90046-6

[5] Nadelhaft I, Booth AM (1984) The location and morphology of preganglionic neurons and the distribution of visceral afferents from the rat pelvic nerve: a horseradish peroxidase study. J Comp Neurol 226 (2) 238–245. 6736301 10.1002/cne.902260207

[6] De Groat WC (1975) Nervous control of the urinary bladder in the cat. Brain Res 87 (2–3) 201–211. 1125771 10.1016/0006-8993(75)90417-5

[7] De Groat WC, Nadelhaft I, Milne RJ, Morgan C, Thor K, et al (1981) Organization of the sacral parasympathetic reflex pathways to the urinary bladder and large intestine. J Auton Nerv Syst 3 (2–4) 135–160. 6268684 10.1016/0165-1838(81)90059-x

[8] Banrezes B, Andrey P, Maschino E, Rampin O, Maurin Y, et al (2002) Spatial segregation within the sacral parasympathetic nucleus of neurons innervating the bladder or the penis of the rat as revealed by three-dimensional reconstruction. Neuroscience 115 (1) 97–109. 12401325 10.1016/s0306-4522(02)00405-0

[9] Carter RR, McCreery DB, Woodford BJ, Bullara LA, Agnew WF (1995) Micturition control by microstimulation of the sacral spinal cord of the cat: Acute studies. IEEE Trans Rehabil Eng 3: 206–214.

[10] De Groat WC (1993) Anatomy and physiology of the lower urinary tract. Urol Clin North Am 20: 383–401. 8351765

[11] De Groat WC (2006) Integrative control of the lower urinary tract: preclinical perspective. Br J Pharmacol 147 (2) 25–40. 10.1038/sj.bjp.0706604 PMC1751498 16465182

[12] Grill WM, Bhadra N, Wang B (1999) Bladder and urethral pressures evoked by microstimulation of the sacral spinal cord in cats. Brain Res 836: 19–30. 10415401 10.1016/s0006-8993(99)01581-4

[13] Tai C, Booth AM, De Groat WC, Roppolo JR (2004) Bladder and urethral sphincter responses evoked by microstimulation of S2 sacral spinal cord in spinal cord intact and chronic spinal cord injured cats. Exp Neurol 190: 171–183. 15473990 10.1016/j.expneurol.2004.07.001

[14] Ding YQ, Kaneko T, Nomura S, Mizuno N (1996) Immunohistochemical localization of mu-opioid receptors in the central nervous system of the rat. J Comp Neurol 367 (3) 375–402. 8698899 10.1002/(SICI)1096-9861(19960408)367:3<375::AID-CNE5>3.0.CO;2-2

[15] Mansour A, Watson SJ (1993) Anatomical distribution of opioid receptors in mammalians: an overview. In: Handbook of experimental pharmacology, Opioids I (Herz A, ed) Springer: Verlag Berlin and Heidelberg GmbH & Co. K. pp. 79–102.

[16] Moriwaki A, Wang JB, Svingos A, van Bockstaele E, Cheng P, et al (1996) mu opiate receptor immunoreactivity in rat central nervous system. Neurochem Res 21 (11) 1315–1331. 8947922 10.1007/BF02532373

[17] Gouardères C, Beaudet A, Zajac JM, Cros J, Quirion R (1991) High resolution radioautographic localization of [125I] FK-33-824-labelled mu opioid receptors in the spinal cord of normal and deafferented rats. Neuroscience 43 (1) 197–209. 1717884 10.1016/0306-4522(91)90427-p

[18] Moskowitz AS, Goodman RR (1984) Light microscopic autoradiographic localization of μ and δ opioid binding sites in the mouse central nervous system. J Neurosci 4 (5) 1331–42. 6327936 10.1523/JNEUROSCI.04-05-01331.1984 PMC6564939

[19] Mansour A, Fox CA, Thompson RC, Akil H, Watson SJ (1994) mu-Opioid receptor mRNA expression in the rat CNS: comparison to mu- receptor binding. Brain Res 643 (1–2) 245–265. 8032920 10.1016/0006-8993(94)90031-0

[20] Dray A, Metsch R (1984) Opioid receptor subtypes involved in the central inhibition of urinary bladder motility. Eur J Pharmacol 104 (1–2) 47–53. 6094211 10.1016/0014-2999(84)90367-4

[21] Dray A, Metsch R (1984) Inhibition of urinary bladder contractions by a spinal action of morphine and other opioids. J Pharmacol Exp Ther 231 (2) 254–260. 6092610

[22] Yoshimura N, Chancellor MB (2007) Physiology and pharmacology of the bladder and urethra (Section XIV, Chapter 56). In: Wein, A.J. (ed) Campbell-Walsh urology vol. 3. 9th edn. Saunders, Philadelphia, P.A. pp. 1922–1972.

[23] Spetea M, Monory K, Tömböly C, Hanoune J, Borsodi A (1998) In vitro binding and signaling profile of the novel mu opioid receptor agonist endomorphin 2 in rat brain membranes. Biochem Biophys Res Commun 250 (3) 720–725. 9784412 10.1006/bbrc.1998.9395

[24] Zadina JE, Hackler L, Ge LJ, Kastin AJ (1997) A potent and selective endogenous agonist for the mu-opiate receptor. Nature 386 (6624) 499–502. 9087409 10.1038/386499a0

[25] Hui R, Wang W, Chen T, Wu SX, Li YQ, et al (2010) Origins of endomorphin-2 immunopositive fibers and terminals in the spinal dorsal horn of the rat. Neuroscience 169: 422–430. 20457220 10.1016/j.neuroscience.2010.05.006

[26] Martin-Schild S, Zadina JE, Gerall AA, Vigh S, Kastin AJ (1997) Localization of endomorphin-2 -like immunoreactivity in the rat medulla and spinal cord. Peptides 18 (10) 1641–1649. 9437728 10.1016/s0196-9781(97)00320-3

[27] Martin-Schild S, Gerall AA, Kastin AJ, Zadina JE (1998) Endomorphin-2 is an endogenous opioid in primary sensory afferent fibers. Peptides 19 (10) 1783–1789. 9880085 10.1016/s0196-9781(98)00136-3

[28] Pierce TL, Grahek MD, Wessendorf MW (1998) Immunoreactivity for endomorphin-2 occurs in primary afferents in rats and monkey. Neuroreport 9 (3) 385–389. 9512376 10.1097/00001756-199802160-00005

[29] Zadina JE, Martin-Schild S, Gerall AA, Ge LJ, Zhang X, et al (1999) Endomorphins: novel endogenous mu-opiate receptor agonists in regions of high mu-opiate receptor density. Ann N Y Acad Sci 897: 136–144. 10676442 10.1111/j.1749-6632.1999.tb07885.x

[30] Zhu C, Hui R, Chen T, Wu SX, Li YQ, et al (2011) Origins of endomorphin-2 immunopositive fibers and terminals in the rat medullary dorsal horn. Brain Res 1410: 38–47. 21813112 10.1016/j.brainres.2011.06.067

[31] Hisamitsu T, De Groat WC (1984) The inhibitory effect of opioid peptides and morphine applied intrathecally and intracerebroventricularly on the micturition reflex in the cat. Brain Res 298 (1) 51–65. 6586255 10.1016/0006-8993(84)91146-6

[32] Gu YM, Chen YC, Ye LM (1991) A new high sensitive HRP-TMB method using Sodium tungstate as a stabilizer: II. Electron microscopy study. Chin J Neuroanat 7 (1) 124–129.

[33] LI JL, Ding YQ, LI YQ, Knaeko T, Mizuno N, et al (1998) Immunohistochemical localization of mu-opioid receptor in primary afferent neurons conatining substnace P or calcitonin gene related peptide: a light and elecrton microscope study in the rat. Barin Res 794 (2) 347–352. 10.1016/s0006-8993(98)00332-1 9622672

[34] De Groat WC, Booth AM, Milne RJ, Roppolo JR (1982) Parasympathetic preganglionic neurons in the sacral spinal cord. J Auton Nerv Syst 5 (1) 23–43. 7056993 10.1016/0165-1838(82)90087-x

[35] Laplane D, Degos JD, Baulac M, Gray F (1981) Bilateral infarction of the anterior cingulate gyri and of the fornices. Report of a case. J Neurol Sci 51 (2) 289–300. 7276980 10.1016/0022-510x(81)90107-6

[36] Blok BF (2002) Brain control of the lower urinary tract. Scand J Urol Nephrol Suppl 210: 11–5. 10.1080/003655902320765908 12475011

[37] Sugaya K, Matsuyama K, Takakusaki K, Mori S (1987) Electrical and chemical stimulations of the pontine micturition center. Neurosci Lett 80 (2) 197–201. 3683977 10.1016/0304-3940(87)90653-7

[38] Beckel JM, Holstege G (2011) Neuroanatomy of the lower urinary tract. Handb Exp Pharmacol 202: 99–116. 10.1007/978-3-642-16499-6_6 21290224

[39] Lagou M, De Vente J, Kirkwood TB, Gillespie JI, Drake MJ, et al (2006) Location of interstitialcells and neurotransmitters in the mouse bladder. BJU Int 97 (6) 1332–1337. 16686734 10.1111/j.1464-410X.2006.06203.x

[40] Wang Y, Fang Q, Lu Y, Li W, Li L, et al (2010) Effects of mechanical stretch on interstitial cells of Cajal in guinea pig bladder. J Surg Res 164 (1) e213–e219 Available: http://www.sciencedirect.com/science/article/pii/S0022480410004270. Accessed 2010 April 4. 20828727 10.1016/j.jss.2010.04.040

[41] Buss RR, Shefchyk SJ (2003) Sacral dorsal horn neurone activity during micturition in the cat. J Physiol 551 (1) 387–396. 12815177 10.1113/jphysiol.2003.041996 PMC2343146

[42] Mannen T (2000) Neuropathological findings of Onuf's nucleus and its significance. Neuropathology 20: 30–33. 10.1046/j.1440-1789.2000.00298.x 11037184

[43] Nagasaka Y, Yokoyama O, Komatsu K, Nakamura Y, Namiki M, et al (2007) Effects of opioid subtypes on detrusor overactivity in rats with cerebral infarction. Int J Urol 14 (3) 226–231. 17430260 10.1111/j.1442-2042.2007.01700.x

[44] Honda CN, Arvidsson U (1995) Immunohistochemical localization of delta- and mu-opioid receptors in primate spinal cord. Neuroreport 6 (7) 1025–1028. 7632887 10.1097/00001756-199505090-00019

[45] Pascual JI, Insausti R, Gonzalo LM (1993) Urinary bladder innervation in male rat: termination of primary afferents in the spinal cord as determined by transganglionic transport of WGA-HRP. J Urol 150 (2 Pt 1) 500–504. 7686986 10.1016/s0022-5347(17)35535-0

[46] Dray A, Nunan L (1985) Opioid inhibition of reflex urinary bladder contractions: dissociation of supraspinal and spinal mechanisms. Brain Res 337 (1) 142–145. 2988707 10.1016/0006-8993(85)91619-1

[47] Dray A, Nunan L (1987) Mu and delta opioid ligands inhibit reflex contractions of the urinary bladder in the rat by different central mechanisms. Neuropharmacology 26 (7A) 753–759. 3627383 10.1016/0028-3908(87)90238-3

[48] Sheldon RJ, Nunan L, Porreca F (1987) Mu antagonist properties of kappa agonists in a model of rat urinary bladder motility in vivo. J Pharmacol Exp Ther 243 (1) 234–240. 2822899

[49] Kontani H, Kawabata Y (1988) A study of morphine-induced urinary retention in anesthetized rats capable of micturition. Jpn J Pharmacol 48: 31–36. 3199606 10.1254/jjp.48.31

[50] Shimizu I, Kawashima K, Ishii D, Oka M (2000) Effects of pentazocine and 1,3-di-o-tolylguanidine (DTG), sigma ligands, on micturition in anaesthetized rats. Br J Pharmacol 131: 610–616. 11015314 10.1038/sj.bjp.0703593 PMC1572351

[51] Igawa Y, Westerling D, Mattiasson A, Andersson KE (1993) Effects of morphine metabolites on micturition in normal, unanaesthetized rats. Br J Pharmacol 110 (1) 257–262. 8220887 10.1111/j.1476-5381.1993.tb13802.x PMC2175977

[52] Jubelin B, Galeano C, Ladouceur D, Lemaire S, Elhilali MM (1984) Effect of enkephalin on the micturition cycle of the cat. Life Sci 34 (21) 2015–2027. 6547198 10.1016/0024-3205(84)90366-7

[53] Okada M, Hisamitsu T (1986) Effects of opiate and opioid peptides administered intrathecally on the pain threshold and micturition reflex in rats. Masui 35 (6) 877–884. 2877104

[54] Maggi CA, Giuliani S, Meli A (1989) Dermorphin inhibits micturition reflex in rats at a central site of action. J Auton Nerv Syst 26 (1) 11–6. 2540230 10.1016/0165-1838(89)90102-1

[55] Farquhar-Smith WP, Rice AS (2001) Administration of endocannabinoids prevents a referred hyperalgesia associated with inflammation of the urinary bladder. Anesthesiology 94: 507–513. 11374613 10.1097/00000542-200103000-00023

[56] Dmitrieva N, Berkley KJ (2002) Contrasting effects of WIN 55212-2 on motility of the rat bladder and uterus. J Neurosci 22: 7147–7153. 12177210 10.1523/JNEUROSCI.22-16-07147.2002 PMC6757898

[57] Hiragata S, Ogawa T, Hayashi Y, Chancellor MB, Yoshimura N, et al (2007) Effects of IP-751, ajulemic acid, on bladder overactivity induced by bladder irritation in rats. Urology 70: 202–208. 17656248 10.1016/j.urology.2007.02.069

[58] Martin RS, Luong LA, Welsh NJ, Martin GR, MacLennan SJ, et al (2000) Effects of cannabinoid receptor agonists on neuronally-evoked contractions of urinary bladder tissues isolated from rat, mouse, pig, dog, monkey and human. Br J Pharmacol 129: 1707–1715. 10780977 10.1038/sj.bjp.0703229 PMC1571997

[59] Pertwee RG, Fernando SR (1996) Evidence for the presence of cannabinoid CB1 receptors in mouse urinary bladder. Br J Pharmacol 118 (8) 2053–2058. 8864542 10.1111/j.1476-5381.1996.tb15643.x PMC1909890

[60] Rivero G, Llorente J, McPherson J, Henderson G, Kelly E, et al (2012) Endomorphin-2: a biased agonist at the μ-opioid receptor. Mol Pharmacol 82 (2) 178–188. 22553358 10.1124/mol.112.078659 PMC3400840

